# Supplementary material for: Integrative Bioinformatic and Epidemiological Analysis of Acetaminophen Use and Risk of Sex Hormone-Related Cancers
Source: Int J Mol Sci. 2025 Dec 29;27(1):376. doi: 10.3390/ijms27010376 (PMC12786045; doi:10.3390/ijms27010376)
Supplement: Supplementary file 1 [file ijms-27-00376-s001.zip › ijms-3990789-supplementary.pdf]

Supplementary Tables

Table S1. Summary of epidemiological studies evaluating the association between acetaminophen use and prostate cancer risk. GPRD (General Practice Research Database); OR (Odds Ratio); CI (Confidence Interval); HR (Hazard Ratio).

Author and year	Study type	Population	Intervention	Main results	Statistical significance	Notes/limitations
Garcia-Rodriguez & Gonzalez-Perez 2004 [32]	Cohort with nested case–control analysis	339,462 men, aged 50–79, GPRD (UK); 2,183 prostate cancer cases and 10,000 controls	Acetaminophen use (by duration: <1 year *vs* >1 year)	>1 year: OR=0.65 (95% CI 0.54–0.78). <1 year: OR=1.14 (95% CI 1.00–1.31)	>1 year: significant risk reduction (95% CI). <1 year: borderline, not significant	No data on OTC drugs; observational results
Jacobs et al. 2011 [31]	Prospective cohort study (CPS-II Nutrition Cohort, USA)	78,485 men, follow-up 1992–2007; 8,092 prostate cancer cases	Regular acetaminophen use (≥30 tablets/month), analysis by duration (<5 years vs ≥5 years)	≥5 years use: RR=0.62 (95% CI 0.44–0.87) overall; RR=0.49 (95% CI 0.27–0.88) aggressive cancer; no effect for <5 years (RR ~1.0)	Significant for ≥5 years use ($p<0.05$, CI <1.0); no significance for shorter use	Low number of long-term users; no dose data; possible confounding
Veitonmäki et al. 2014 [33]	Prospective cohort study within Finnish Prostate Cancer Screening Trial (ERSPC)	78,615 men, aged 55–67, Finland; follow-up 1996–2009	Acetaminophen use (national prescription registry + OTC survey)	Current use: HR=1.51 (95% CI 1.25–1.81, screening arm); HR=1.65 (95% CI 1.44–1.90, control arm). Risk highest for metastatic cancer: HR=3.44 (95% CI 2.60–4.55)	Significantly increased risk (95% CI)	No dose–response relationship observed; ethnically homogeneous Finnish population
Walter et al. 2011 [35]	Prospective cohort study (VITamins and Lifestyle, USA)	30,782 men, aged 50–76; follow-up 2000–2008	Acetaminophen ≥1 day/week for ≥1 year; categories: none, low, high (≥4 days/week and ≥4 years)	Overall prostate cancer: HR=1.00 (95% CI 0.73–1.37); Aggressive prostate cancer: HR=0.74 (95% CI 0.45–1.21)	Neither result is statistically significant	Self-reported exposure; no precise dose information
Murad et al. 2011 [36]	Cross-sectional, case–control study within ProtecT (UK)	1,016 prostate cancer cases and 5,043 controls; men aged 50–69	Self-reported acetaminophen use (questionnaire, OTC + prescription)	OR=1.20 (95% CI 0.90–1.60)	Not statistically significant	Self-reported exposure; possible underestimation of OTC impact

Table S2. Overview of studies on acetaminophen use and ovarian cancer risk and survival, including study type, effect estimates, and main conclusions. RR (Relative Risk); OR (Odds Ratio); HR (Hazard Ratio); CI (Confidence Interval).

Author and year	Study type	Population	Intervention	Main results	Statistical significance	Notes/limitations
Bonovas et al. 2006 [60]	Meta-analysis (8 studies: 4 cohort, 4 case–control)	8 observational studies published 1998–2004; sample sizes ranged from thousands to >600,000 women	Acetaminophen use (regular vs occasional, <10 years vs >10 years)	Overall: RR=0.84 (95% CI 0.70–1.00). Case–control studies: RR=0.69 (95% CI 0.52–0.93). Cohort studies: RR=0.97 (95% CI 0.85–1.11). Regular use: RR=0.70 (95% CI 0.51–0.95)	The protective effect is significant in case–control studies and with regular use; borderline significance in overall analysis; no significance in cohort studies or with >10 years of use	Heterogeneous exposure definitions; most studies from the USA
Trabert et al. 2014 [57]	Pooled analysis (12 population-based case–control studies, Ovarian Cancer Association Consortium)	7,776 invasive ovarian cancer cases and 11,843 controls; women from Australia, USA, UK, and Denmark	Regular acetaminophen use: analysis by frequency, dose, and duration	Acetaminophen: OR=0.99 (95% CI 0.88–1.12)	No significant association for acetaminophen	Self-reported medication use
Dixon et al. 2017 [67]	Pooled analysis (12 case–control studies, Ovarian Cancer Association Consortium)	7,694 women with invasive epithelial ovarian cancer	Self-reported pre-diagnosis acetaminophen use (regular = ≥1 time/week)	HR=1.01 (95% CI 0.93–1.10)	No significant associations for acetaminophen	Pre-diagnosis exposure only, no post-diagnosis data
Fairfield et al. 2002 [56]	Prospective cohort study (Nurses' Health Study, USA)	76,821 women, follow-up 1976–1996; 333 invasive ovarian cancer cases	Acetaminophen use (frequency and duration; ≥5 days/month)	≥5 days/month: RR=0.81 (95% CI 0.46–1.43)	Not statistically significant	Small number of acetaminophen users; self-reported data; no information on reasons for use; mostly Caucasian participants
Barnard et al. 2018 [58]	Prospective cohort study (Nurses' Health Study I & II, USA)	205,498 women (NHS: 93,664, NHSII: 111,834); follow-up 1980–2015; 1,054 ovarian cancer cases	Regular acetaminophen use; exposure updated every 2–4 years	HR=1.02 (95% CI 0.86–1.21)	No significant associations for standard-dose of acetaminophen	Self-reported exposure
Trabert et al. 2019 [65]	Prospective cohort analysis (Ovarian Cancer Cohort	758,829 women; 3,514 ovarian cancer cases; mean follow-up 10.8 years	Self-reported acetaminophen use (frequency, dose, duration)	Frequent use: HR=1.05 (95% CI 0.88–1.24). Daily use: HR=1.28 (95% CI	Daily use: significant increased risk (95% CI); other results are not significant	Self-reported data; no data on indications for use

	Consortium, 13 studies)			1.00–1.65). For serous cancer: HR=1.70 (95% CI 1.14–2.55)		
Baandrup et al. 2014 [62]	Nationwide nested case–control study (Denmark)	3,471 epithelial ovarian cancer cases and 50,576 controls; women aged 30–84 years; source population ~2.7 million women	Prescription acetaminophen: analyses by use, duration (<5, 5–10, >10 years), and intensity (low, medium, high)	Ever use: OR=0.82 (95% CI 0.74–0.92). Serous cancer: OR=0.82 (95% CI 0.72–0.94). Long-term (≥10 years), high-intensity use: OR=0.45 (95% CI 0.24–0.86); for serous cancer OR=0.37 (95% CI 0.16–0.84)	Significant risk reduction with acetaminophen use, particularly long-term and high-intensity	No OTC data (approx. 40–45% of acetaminophen in Denmark sold OTC)
Baandrup 2015 [63]	Nationwide registry-based case–control study (Denmark)	3,471 epithelial ovarian cancer cases (2000–2009) and 50,576 controls; women aged 30–84 years; nationwide registry data	Prescription acetaminophen, ≥2 prescriptions (analyses by duration: <5, 5–10, >10 years; intensity: low, medium, high; continuous use ≥5 years)	Ever use: OR=0.82 (95% CI 0.74–0.92). Strongest risk reduction with long-term (>10 years), high-intensity use: OR=0.45 (95% CI 0.24–0.86). Continuous use ≥5 years: OR=0.73 (95% CI 0.56–0.96)	All results reported as 95% CI	No OTC data (approx. 40–45% of acetaminophen in Denmark sold OTC)
Pinheiro et al. 2009 [55]	Prospective cohort study (Nurses' Health Study I & II, USA)	197,486 women (84,303 NHS, 113,183 NHS-II); follow-up 24 years (NHS) and 16 years (NHS-II); 666 ovarian cancer cases	Regular acetaminophen use (≥2 times/week), analyzed by frequency and duration	Regular use: HR=1.14 (95% CI 0.92–1.43). No association with frequency (≥5 days/week: HR=1.13, 95% CI 0.70–1.82) or duration (≥5 years: HR=0.86, 95% CI 0.55–1.37)	Not statistically significant for all categories of use	Self-reported exposure; mostly Caucasian participants; possible underestimation for sporadic use
Nagle et al. 2015 [68]	Prospective cohort survival study	1305 women with invasive epithelial ovarian cancer (Australia); mean follow-up 4.9 years; 834 deaths (779 from ovarian cancer)	Self-reported acetaminophen use in the 5 years prior to diagnosis (frequency: never, occasional, ≤1/week, >1/week)	Any use: HR=0.91 (95% CI 0.69–1.20). Occasional: HR=0.91 (95% CI 0.69–1.20). ≤1/week: HR=0.92 (95% CI 0.68–1.24). >1/week: HR=0.91 (95% CI 0.67–1.26)	Not statistically significant	Only pre-diagnostic use assessed, no post-diagnostic data; self-reported exposure (possible recall bias)
Schildkraut et al. 2006 [64]	Population-based case–control study (North	586 ovarian cancer cases and 627 controls;	Acetaminophen use ≥3 months during the 5 years	Any acetaminophen use: OR=0.78 (95%	Not statistically significant for all categories of use	Questionnaire-based data (recall bias possible);

	Carolina Ovarian Cancer Study, USA)	women aged 20–74 years; recruitment 1999–2003, North Carolina	prior to diagnosis (cases) or interview (controls)	CI 0.56–1.08). Frequent use ≥8 times/month <3 years: OR=0.62 (95% CI 0.36–1.08). ≥8 times/month ≥3 years: OR=0.77 (95% CI 0.42–1.41)		limited assessment of long-term use; exposure window restricted to 5 years before diagnosis

Table S3. Summary of studies evaluating the association between acetaminophen use and endometrial cancer risk. RR (Relative Risk); OR (Odds Ratio); HR (Hazard Ratio); CI (Confidence Interval); MV – Multivariable.

Author and year	Study type	Population	Intervention	Main results	Statistical significance	Notes/limitations
Moysich et al. 2005 [72]	Hospital-based case–control study	427 endometrial cancer cases and 427 controls (women without neoplasms, hospitalized for other reasons); mean age ~62 years	Regular acetaminophen use (≥1 time/week for ≥6 months); analyses by frequency, duration, BMI	Regular use: OR=0.96 (95% CI 0.60–1.54). 1–6 times/week: OR=0.92 (95% CI 0.55–1.56). ≥7 times/week: OR=1.12 (95% CI 0.42–2.98). 1–10 years use: OR=1.55 (95% CI 0.65–2.05). >10 years: OR=0.69 (95% CI 0.33–1.46). Cumulative use >10 tablet-years: OR=0.49 (95% CI 0.15–1.60)	No statistically significant associations in the overall population. BMI-stratified analysis: no association among obese women	Self-reported exposure (possible recall bias); limited statistical power in subgroups
Neill et al. 2012 [75]	Nationwide case–control study (Australia)	1,398 endometrial cancer cases and 740 controls; women aged 18–79	Acetaminophen use during the past 5 years (frequency: occasional, ≤1/week, ≥2/week)	Ever use: OR=1.19 (95% CI 0.86–1.65). Occasional: OR=1.40 (95% CI 0.99–2.00). ≤1/week: OR=0.99 (95% CI 0.70–1.41). ≥2/week: OR=1.23 (95% CI 0.80–1.90)	Not statistically significant	Self-reported exposure (possible recall bias); no dose data
Viswanathan et al. 2008 [73]	Prospective cohort study (Nurses' Health Study, USA)	82,971 women; follow-up 1980–2004; 747 invasive endometrial cancer cases	Regular acetaminophen use (since 1990; ≥1 time/week)	MV RR for use 1–7 days/week: 0.86–1.21 (all 95% CIs include 1.0). No differences by frequency (1, 2–3, 4–5, 6–7 days/week)	Not statistically significant	Analysis limited to 1990–2004; relatively small case numbers in subgroups; self-reported exposure; no detailed dose data
Ding et al. 2017 [76]	Systematic review and meta-analysis of observational studies (7 studies: 4 cohort, 3 case–control)	3,874 endometrial cancer cases; cohort studies with populations ranging from 26,272 to 82,971	Acetaminophen use (ever vs never; highest frequency/duration vs never)	Ever use vs never use: RR=1.02 (95% CI 0.93–1.13). Highest frequency/duration *vs* never use:	No statistically significant associations	Mostly self-reported data; no dose–response analysis possible

		women; majority in USA, one in Australia, one in Denmark		RR=0.88 (95% CI 0.70–1.11)		
Webb et al. 2019 [77]	Pooled analysis: 7 cohort + 5 case–control studies	7,120 endometrial cancer cases and 16,069 controls; women from the USA, Europe, and Australia	Regular acetaminophen use (≥1 time/week)	No overall association: OR=0.97 (95% CI 0.87–1.07). By BMI: overweight women OR=0.79 (95% CI 0.64–0.96); normal weight OR=1.10 (95% CI 0.91–1.33); obese OR=1.04 (95% CI 0.86–1.24)	No significance overall	Mostly self-reported data (except Sweden – prescription registries); No dose or frequency detail in most studies

Table S4. Summary of epidemiological studies evaluating the association between acetaminophen use and breast cancer risk. RR (Relative Risk); OR (Odds Ratio); HR (Hazard Ratio); CI (Confidence Interval); IRR (Incidence Rate Ratio).

Author and year	Study type	Population	Intervention	Main results	Statistical significance	Notes/limitations
Friis et al. 2008 [78]	Prospective cohort study (Danish Diet, Cancer and Health Cohort)	28,695 women, aged 50–64, 847 breast cancer cases	Self-reported acetaminophen use in questionnaire (≥2 tablets/month) plus prescription registry data (1995–2003)	Acetaminophen users: RR=1.04 (95% CI 0.86–1.26). By frequency: 2–3 tablets/month: RR=1.09 (95% CI 0.87–1.37); 1–6 tablets/week: RR=1.07 (95% CI 0.81–1.41); 1–6 tablets/day: RR=0.76 (95% CI 0.46–1.26). Prescription-based analysis: RR=1.02 (95% CI 0.77–1.36)	Not statistically significant	No dose data; possible recall bias;
Kwan et al. 2007 [79]	Prospective cohort study	2,292 women with early-stage breast cancer (stage I–IIIa), diagnosed 1997–2000;	Regular acetaminophen use (≥3 days/week); analysis: never, post-diagnosis only, pre- and post-diagnosis	Ever use: RR=1.21 (95% CI 0.73–2.00). Post-diagnosis: RR=1.49 (95% CI 0.76–2.93). Pre- and post-diagnosis: RR=1.00 (95% CI 0.49–2.04)	Not statistically significant	Self-reported exposure; no dose/frequency details; small number of acetaminophen users
Eliassen et al. 2009 [81]	Prospective cohort study (Nurses' Health Study II, USA)	112,292 premenopausal women; follow-up 1989–2003; 1,345 invasive breast cancer cases	Regular acetaminophen use (≥2 times/week); analyses by frequency, dose, duration, ER/PR status	Current use: RR=0.99 (95% CI 0.84–1.16). <5 years: RR=0.97 (95% CI 0.81–1.16). ≥5 years: RR=1.07 (95% CI 0.78–1.47). ER+/PR+: RR=0.98 (95% CI 0.78–1.22). ER–/PR–: RR=1.00 (95% CI 0.67–1.47)	Not statistically significant	Self-reported exposure; no dose data in early years; possible recall bias

Zhang et al. 2012 [82]	Prospective cohort study (Nurses' Health Study, USA)	84,602 postmenopausal women, cancer-free in 1980; follow-up until 2008; 4,734 breast cancer cases	Regular acetaminophen use (since 1990; dose, frequency, duration)	Current use – RR=0.89 (95% CI 0.83–0.96)	Statistically significant reduced risk overall (95% CI)	Self-reported exposure (possible recall bias)
Harris et al. 2003 [87]	Prospective cohort study (Women's Health Initiative Observational Study, USA)	80,741 postmenopausal women, aged 50–79; mean follow-up 43 months; 1,392 confirmed breast cancer cases	Regular acetaminophen use (≥2 tablets/week); analyzed by duration (1–4 years, ≥5 years)	1–4 years: RR=1.02 (95% CI 0.75–1.37). ≥5 years: RR=0.96 (95% CI 0.76–1.20)	Not statistically significant	Data collected by questionnaire and validated with pill bottle labels; no info on frequency/compli ance; exposure only at baseline.
Bosco et al. 2011 [96]	Prospective cohort study (Black Women's Health Study, USA)	53,151 African American women; follow-up 1995–2007; 1,275 breast cancer cases	Regular acetaminophen use (≥3 days/week); baseline and time-varying analyses; by duration (<1 year, 1–4 years, ≥5 years)	Baseline use: IRR=0.90 (95% CI 0.76–1.07). Time-varying analysis: current use IRR=0.80 (95% CI 0.65–0.98). Long-term use (≥5 years): IRR=0.70 (95% CI 0.51–0.97)	Significant risk reduction for current and ≥5 years of use (95% CI)	Self-reported data; no dose information;
Brasky et al. 2010 [80]	Population-based case–control study	1,170 breast cancer cases and 2,115 controls; women aged 35–79; recruitment 1996–2001	Acetaminophen use in the year prior to interview; analyzed by frequency and intensity	Any acetaminophen use: OR=0.97 (95% CI 0.83–1.15). Infrequent users (≤14 days/month): OR=0.96 (95% CI 0.81–1.14). Regular users (>14 days/month): OR=0.98 (95% CI 0.66–1.46). Low intensity (<2 pills/day): OR=0.86 (95% CI 0.66–1.13). High intensity (≥2 pills/day): OR=1.01 (95% CI 0.85–1.20)	Not statistically significant	Self-reported exposure; no dose/duration details; possible recall bias
de Pedro et al. 2015 [84]	Systematic review and meta-analysis of observational studies	49 studies (23 case–control, 24 cohort, 2 RCT)	Acetaminophen use (ever vs never use; highest dose/duration)	Case–control: OR=0.85 (95% CI 0.76–0.95). Cohort: RR=0.95 (95% CI 0.88–1.01)	Case–control: significant protective effect (95% CI)	
